# Effect of Different Levels of Energy Diet Restriction on Energy Balance, Leptin and CL Development, Vascularization, and Function in South American Camelids

**DOI:** 10.3389/fvets.2020.598147

**Published:** 2020-12-16

**Authors:** Cecilia Norambuena, Francisca Hernandez, Jorge Alfaro, Gonzalo Silva, Shirley Topp, Marcelo Ratto

**Affiliations:** ^1^Department of Veterinary Science and Public Health, Faculty of Natural Resources, Catholic University of Temuco, Temuco, Chile; ^2^Nucleus of Research in Food Production, Faculty of Natural Resources, Catholic University of Temuco, Temuco, Chile; ^3^Institute of Animal Science, Faculty of Veterinary Science, Austral University, Valdivia, Chile

**Keywords:** diet restriction, leptin, corpus luteum, llama, alpaca, progesterone

## Abstract

The objective was to determine the effect of energy diet restriction on energy balance, systemic leptin and corpus luteum (CL) vascularization, development, and function in South American camelids. In experiment 1, adult llamas were randomly assigned to receive a diet of 70% of their maintenance energy requirements (MER) (Restricted group, *n* = 7) or fed *ad libitum* (Control group, *n* = 7) during 28 days. Body live weight (BLW) and body condition score (BCS) were recorded, blood samples were collected every 2 weeks to measure plasma leptin concentrations, and energy metabolites were quantified. In experiment 2, adult alpacas were randomly assigned to receive a diet of 40% MER for 21 days (Restricted group, *n* = 7) or fed *ad libitum* (Control group, *n* = 7). Then, ovulation was induced with gonadorelin acetate (day = 0), and trans-rectal ultrasonography (7.5 MHz) was performed using B and Doppler mode to record the diameter of the pre-ovulatory follicle, ovulation, CL diameter, and vascularization from Days 0 to 13. Blood samples were collected every 48 h from Days 1 to 13 to quantify plasma leptin and progesterone concentrations. In experiment 1, energy diet restriction of 70% MER did not affect plasma leptin concentration and metabolic parameters of the Restricted group. In experiment 2, the Restricted group had a lower BCS (*p* < 0.001), a smaller diameter of the CL on Days 5 and 7 (*p* < 0.05), and a smaller maximum diameter of the CL (10.2 ± 0.6 mm) than the Control group (12.1 ± 0.6 mm; *p* = 0.04). Low energy restriction of 70% MER for 28 days did not affect the energy balance of llamas (Experiment 1). Moderate energy restriction of 40% MER for 21 days negatively affected energy balance (BCS), and CL development but not its vascularization, leptin, and progesterone concentrations. These species must be submitted to longer periods or a higher level of energy restriction to impair ovarian function.

## Introduction

Members of the Camelidae family are recognized for their ability to survive and reproduce in extreme environments (1). In Chile, the arrival of ruminants during the Spanish conquest decimated the population of camelids and displaced the domestic (*Lama glama, Vicugna pacos*) and wild species (*Vicugna vicugna, Lama guanicoe*) toward the marginal lands of the country (2, 3). Low fertility rates of highland camelid herds have been described [about 50%, (4)]. In a previous alpaca study (5), we have documented an overall embryo loss of 45.4% (49/108), during the 35 days after mating, which was close to the 50–58% reported in earlier studies (4, 6). Although the main factors related to embryo loss in these species are unknown, the poor nutritional status of the High Andean herds could be related (7).

Leptin, an adipocyte-derived hormone, acts as a critical metabolic signal linking nutrition and reproductive function (8). The rapid decrease in leptinemia in underfed animals could be translated as an acute signal to stimulate re-feeding behavior and glucocorticoid secretion; to decrease thyroid activity, energy expenditure, insulin sensitivity, and protein synthesis; and to block reproduction (9). Leptin modulates GnRH secretion in the hypothalamus indirectly through stimulation of neuropeptide Y and kisspeptin (10, 11). Systemic administration of leptin increased gonadotropin secretion in fasted ruminants and rats (12, 13). Leptin receptor, OB-R, has been characterized in the granulosa and theca cells, luteal cells, the ovarian stroma, and in endothelial cells in various species (14, 15) and recently in alpacas (16). These evidences support the notion that leptin may have a potential role in the maturation and selection of developing follicles, corpus luteum (CL) formation, function, and regression during estrus cycle via an autocrine/paracrine mechanism in several species (15).

In a previous study (17), severe long-term nutritional restriction negatively affected leptin concentration, the diameter of the pre-ovulatory follicle, and corpus luteum (CL) and progesterone production in llamas. The day-by-day profile of plasma leptin concentration was correlated with the CL growth during the entire luteal phase suggesting a potential local role of leptin on CL development in llamas. Kumar et al. (18) demonstrated that the leptin mRNA highest levels were in mid and late luteal stages consistent with *in vivo* luteinization of buffalo CL and declined coincidental to luteal regression. Also, the increased expression of steroidogenic enzymes (StAR, P450scc, HSD) has been correlated with the OB gene and OBR receptor activity in mid luteal phase suggesting a key role of leptin in progesterone production (18).

The mechanism of action of leptin on CL development is unknown. Wiles et al. (19) have demonstrated that the addition of leptin in an *in vitro* culture of goats luteal cells increased the expression of angiogenic factors such as angiotensin I (Ang1), fibroblast growth factor 2 (FGF2), and vascular endothelial growth factor (VEGF). In fact, CL vascularization did increase in alpacas previously treated with leptin during follicular phase (pre-ovulatory follicle > 7 mm of diameter) and given GnRH for ovulation induction, however, CL diameter, plasma luteinizing hormone (LH), and progesterone concentration were similar between leptin and non-leptin-treated females (20).

The objectives of this study were to determine the effect of low energy diet restriction on energy balance in llamas (Experiment 1) and to evaluate the effect of moderate energy restriction on energy balance on leptin concentration and CL vascularization, development, and function in alpacas (Experiment 2).

## Materials and Methods

Experimental procedures were reviewed and approved by the Bioethical Committees of the Universidad Austral de Chile and Universidad Católica de Temuco and were performed in accordance with the animal care protocols established by the same institutions and in accordance with Chilean Animal Protection Act (2009).

### Experiment 1: Effect of Low Energy Diet Restriction (70% of the Maintenance Energy Requirements) on Energy Balance in Llamas

#### Animals and Nutrition Management

This study was conducted in the llama farm at the University Austral of Chile located in Valdivia (39°38′S, 72°35′W). Mature non-lactating, non-pregnant, female llamas (*n* = 14), 6–9 years old, with a body condition score (BCS) of 3–3.5 [scale 1–5, (21)], were randomly assigned to an energy Restricted or a Control group (*n* = 7 per group). The Control group, weighting 149 ± 6 kg (mean ± SEM), was fed *ad libitum* with *Ballica* sp. hay [4.4% crude protein (CP); 5.7% total ashes (TA), and 1.8 Mcal/kg metabolic energy (ME), on dry matter base] and 300 g day^−1^ of commercial pelleted concentrate (16% CP; 5.4% TA, and 3.0 Mcal/kg ME; Cosetán®, Suralim, Osorno). Llamas of the Restricted group, weighting 146 ± 4 (mean ± SEM), were individually fed with the same diet as that of the Control group (3 hay:1 pellet, on the basis of total energy intake), but the amount of food provided 70% of maintenance energy requirements (MER) of the initial body live weigh (BLW). MER was calculated for each female using the following equation: 61.2 ^*^ BLW^0.75^ (22). Llamas were kept indoors and fed under nutritional management during 28 days.

Llamas received water and mineral salts (Usablock®, Sweetlix) *ad libitum*, and they were dewormed using Sofomax® (Intervet). BLW (kg) and BCS were assessed weekly. Blood samples (4 ml) were collected by jugular venipuncture into heparinized tubes every 2 weeks to determine metabolic markers and plasma leptin concentration. Plasma was separated by centrifugation at 1,500*g* for 15 min and stored at −80°C until assayed.

#### Metabolic Markers

Metabolites such as glucose, triglycerides, non-esterified fatty acids, beta-hydroxybutyrate, cholesterol, and urea were analyzed as previously described (17, 23). In brief, glucose concentration was measured using a portable equipment (One touch®, LifescanInc, USA). Triglycerides plasma concentration (GPO-PAD enzymatic method), non-esterified fatty acids (NEFA; ASC-ACOD enzymatic colorimetric method), beta-hydroxibutirato (BHB; 3 HBDH dependent enzymatic method), cholesterol (CHOD-PAD enzymatic method), and urea (kinetic glutamic-dehydrogenase method) were determined using Cobas-Miras-Plus® autoanalyzer (Roche D-10587, Berlin, Germany).

#### Leptin Concentration Analysis

Plasma leptin concentration was determined by radioimmunoassay (RIA; multi-species leptin kit®, Millipore, USA), using a 1470 Wallac Wizard Gamma counter (PerkinElmer Inc, USA). The limit of sensitivity and intra-assay coefficient of variation were 2.5 ng/ml and 10%, respectively.

### Experiment 2: Effect of Moderate Energy Diet Restriction (40% of the Maintenance Energy Requirements) on Energy Balance and Luteal Function in Alpacas

#### Animals, Nutrition Management, and Experimental Design

**Figure 1:**
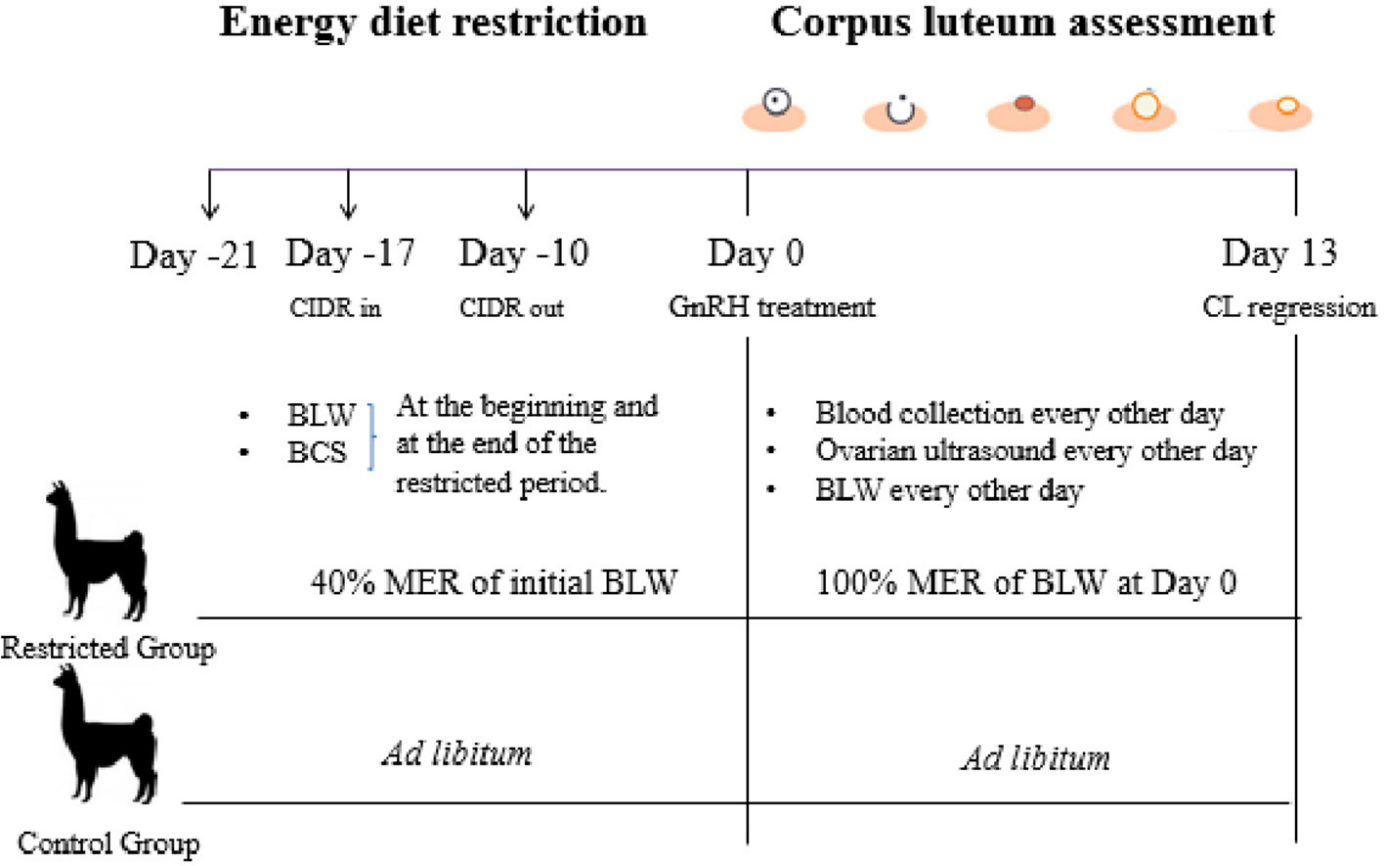
Experimental design of Experiment 2.

The experiment was conducted in the alpaca farm at the Universidad Católica de Temuco, located in Temuco (38°46′S, 72°38′W). Non-pregnant non-lactating adult alpacas (*n* = 14) aged 3 to 9 years old with a BCS of 3 to 3.5 (scale 1–5; 20) were used in the study. Females were randomly assigned to the Restricted or Control group (*n* = 7 per group). The Control group weighting was fed *ad libitum* with *Ballica* sp. hay (4.5% CP, 1.2% TA, 2.6 Mcal/kg ME, on dry matter base) and 200 g day^−1^ of commercial pelleted concentrate (17% CP, 6% TA, 3 Mcal/kg ME on dry matter base; Cosetán®, Iansagro SA, Chile). Alpacas in the Restricted group were individually fed twice daily, with hay and pellets (3:1 on the basis of total energy intake), but the amount of food was reduced to 40% MER of each alpaca. The MER was calculated using the equation 66^*^ initial BLW^0.75^ (7). Alpacas were kept indoors and fed under nutritional management for 21 days (Day −21 to Day −1, Figure 1). They received water and mineral salts (Usablock®, Sweetlix) *ad libitum*, and they were dewormed using Sofomax® (Intervet). On the fourth day after the starting of the diet, alpacas received an intravaginal progesterone device (CIDR-B®, Easy-Breed, 0.3 g, Lab. Pfizer NZ) for 7 days to synchronize follicular wave emergence as previously described (24). All alpacas were examined daily by transrectal ultrasonography using a 7.5 MHz linear array transducer coupled to a monitor (Sonovet r3, Samsung Madison, USA) after the day of progesterone removal to determine the diameter of the pre-ovulatory follicle. Ovulation was induced 10 days after progesterone removal with an intramuscular administration of 50 μg of gonadorelin acetate (GnRH, Gonasyl®, Virbac, Day 0 = GnRH treatment, Figure 1). The ovaries were examined every other day from Day 1 to Day 13 to determine ovulation, CL diameter, and vascularization using B and Doppler mode. Ovulation was defined as the disappearance of a large follicle (≥7 mm) that had been detected in the previous examination.

Alpacas from the Restricted group were switched from 40% MER of the initial BLW to 100% MER of the BLW recorded at Day 0 (GnRH treatment, see Figure 1). This change of diet restriction was conducted in order to maintain the new BLW recorder at Day 0 until Day 13 during CL assessment. BLW and BCS were recorded twice in alpacas of both groups, at the beginning and at the end of the restricted period. BLW was recorded every other day from Day 1 to Day 13 using a digital scale (±0.1 kg).

#### B and Power Doppler Ultrasonography and Image Analysis

CL diameter and vascularization were recorded using B and Power Doppler mode from Day 1 to Day 13 by an operator who was blinded to the treatments and according to previous South American camelid studies (25, 26). In brief, the echotexture of the CL was measured in B mode at its maximum diameter using horizontal and vertical calipers. In Power Doppler mode, cineloops (10 s in length) of the CL vascularization were recorded during Power Doppler imaging and downloaded into VLC media player (www.videolan.org, Version 2.0, Boston, USA). Cineloops were examined frame by frame to select three images that represented the maximum vascular signal at the maximum cross-sectional area of the CL. Images were saved in JPG format with minimal compression and analyzed by ImageJ software (National Institute of Health, Maryland, Washington DC, USA) to determine the area of vascularization of the CL. The area of vascularization was estimated by measuring the area (cm^2^) of the vascular flow signals (Power-Doppler) overlaying the B-mode image of the CL; the average of the three images was taken as the value for a given animal on a given day.

#### Leptin and Progesterone Concentration Analysis

Blood samples (4 ml) were collected into heparinized tubes to determine plasma leptin and progesterone concentration from Day 1 to Day 11 (Day 0 = GnRH treatment) and Day 1 to Day 13, respectively. Plasma leptin concentration was determined using the Sheep Leptin ELISA Kit® (MyBiosource, USA) which was validated previously (20). In brief, serially diluted plasma from alpacas containing high concentrations of leptin produced a displacement curve parallel to the standard curve. Intra- and inter-coefficients of variation were 5.0 and 7.2%, respectively, and the limit of sensitivity was 2.5 ng/ml. Plasma progesterone concentration was determined using a commercial, double-antibody radioimmunoassay kit (COAT-A COUNT 17A-OH Progesterone, Siemens, USA). The intra-assay coefficient of variation varied between 0.5 and 3.5%, and the inter-assay coefficient of variation was between 0.5 and 2.1%. The limit of sensitivity was 0.61 ng/ml. Analyses were performed at the Universidad de Concepción, Chile.

### Statistical Analyses

Normality and homoscedasticity were assessed by Kolmogorov-Smirnov and Levene test, respectively, Log_10−_ transformations were performed whenever necessary. Paired or unpaired Student *t* test were used to compare non-serial data as BLW, BCS, pre-ovulatory follicle diameter, and maximum CL diameter between groups. A mixed ANOVA with time points as repeated measurements was used to compare serial data (BLW, leptin and progesterone plasma concentration, CL diameter, and vascularization area). If significant main effects or interactions were detected (*p* < 0.05), ANOVA pairwise comparisons with Bonferroni adjustment were used. Pearson's correlation was used to determine relationships between CL diameter, vascularization area, and plasma progesterone concentration. Statistical analyses were done using SPSS Program (V 20). Data are reported as mean ± SEM.

## Results

### Experiment 1

Plasma leptin concentration, metabolite markers, BLW, and BCS did not differ between the Restricted and Control groups (Table 1). The initial and final BLW and BCS of the Restricted group did not differ (*p* = 0.06 and *p* = 0.9, respectively). The Restricted group lost 9 kg, equivalent to 6% of its initial BLW. Initial and final BLW and BCS of Control group also were similar (*p* = 0.4 and *p* = 0.9, respectively).

**Table 1:** Plasma leptin concentration, metabolites, body weight and body condition score (Mean ± SEM) of llamas fed 70% energy of maintenance requirements (Restricted group; *n* = 7), or fed *ad libitum* (Control group, *n* = 7) during 28 days.

**Variable**	**Day 1**			**Day 14**			**Day 28**		
	**Restricted**	**Control**	***P***	**Restricted**	**Control**	***P***	**Restricted**	**Control**	***P***
Leptin (ng/mL)	7.7 ± 1.4	5.5 ± 0.5	0.7	5.3 ± 0.4	7.5 ± 1.6	0.6	6.7 ± 1.2	5.5 ± 1.4	0.6
Glucose (mg/dL)	145 ± 5	140 ± 4.8	0.1	138 ± 5.9	151 ± 4.9	0.1	152 ± 4.6	142 ± 4.2	0.3
BHB (mmol/L)	0.07 ± 0.04	0.05 ± 0.03	0.8	0.004 ± 0.004	0.003 ± 0.03	0.3	0.07 ± 0.04	0.05 ± 0.04	0.7
NEFA (mmol/L)	0.05 ± 0.01	0.08 ± 0.01	0.3	0.17 ±0.03	0.09 ± 0.03	0.09	0.17 ± 0.03	0.2 ± 0.03	0.2
Cholesterol (mmol/L)	1.5 ± 0.4	1.8 ± 0.4	0.3	2.2 ± 0.4	2.6 ± 0.5	0.6	1.5 ± 0.3	1.8 ± 0.4	0.6
Urea (mmol/L)	7.9 ± 0.4	7.4 ± 0.4	0.6	5.7 ± 0.6	6.9 ± 0.7	0.2	6.2 ± 0.7	6.2 ± 0.7	0.9
Triglycerides (mml/L)	0.3 ± 0.05	0.4 ± 0.07	0.2	0.3 ± 0.06	0.4 ± 0.1	0.6	0.4 ± 0.05	0.5 ± 0.07	0.8
Total Protein (g/L)	101 ± 7.1	103 ± 2.4	0.9	69 ± 3.6	77 ± 2.4	0.1	73 ± 2.3	84 ± 5.2	0.06
BLW (kg)	146 ± 3.8	149 ± 6.2	0.7	137 ± 3.8	147 ± 6.5	0.7	137 ± 3.3	146 ± 4.8	0.2
BCS (1–5)	3.5 ± 0.1	3.5 ± 0.2	0.4	3.4 ± 0.1	3.5 ± 0.2	0.4	3.2 ± 0.1	3.5 ± 0.1	0.3

*NEFA is non-esterified fatty acids; BLW is body live weight; BCS is Body condition score*.

### Experiment 2

**Figure 2:**
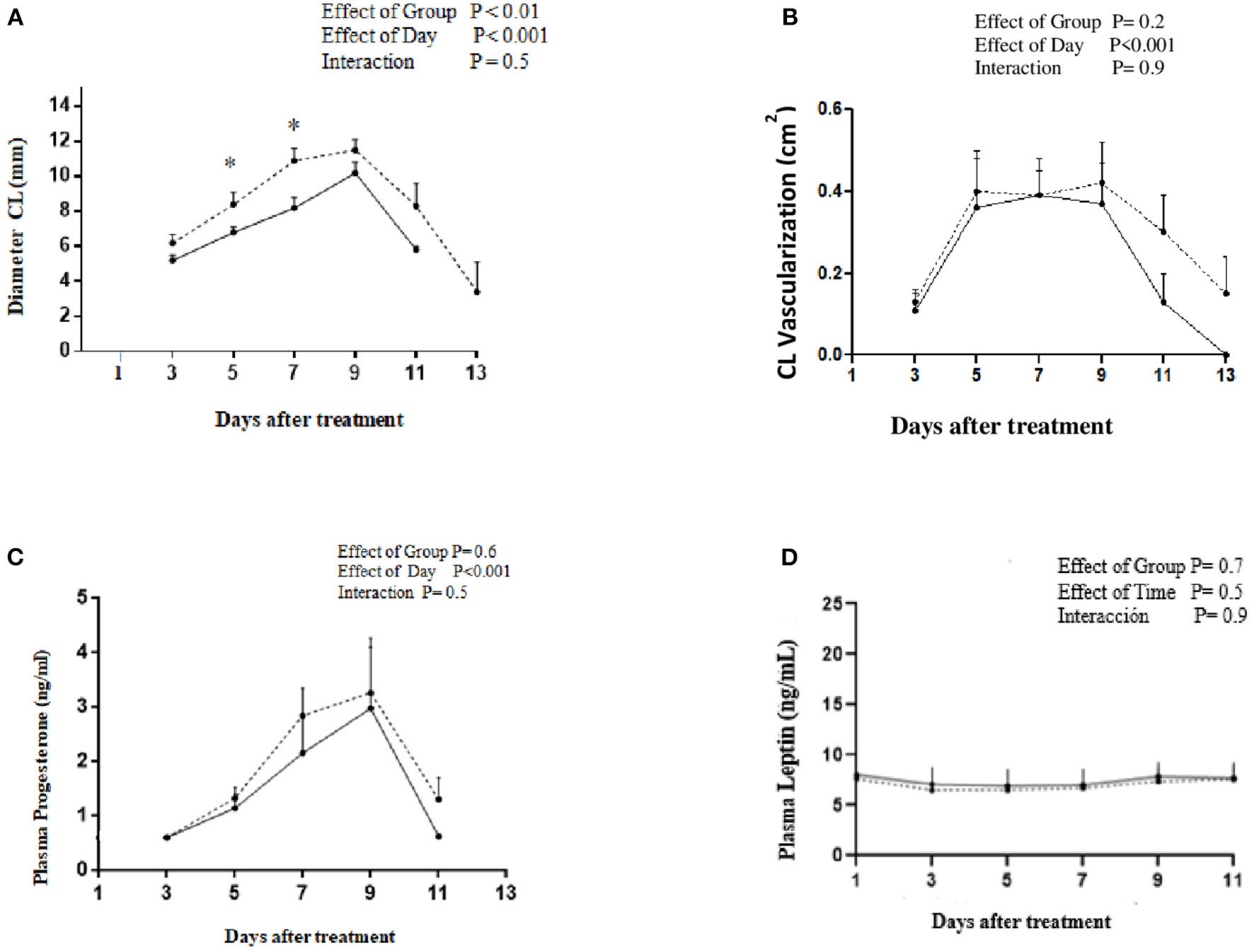
Profiles of **(A)** CL diameter, **(B)** CL vascularization, **(C)** plasma progesterone concentration, and **(D)** plasma leptin concentration after treatment with GnRH to induce ovulation in alpacas under energy restriction 40% (______, *n* = 7) or fed *ad libitum* (-------, *n* = 7).

The Restricted and Control groups had similar BLW and BCS at the beginning of the diet restricted period (Table 2). Although BLW was not different between groups, BCS was higher in the Control than that of the Restricted group after 21 days of 40% MER. The Restricted group reduced the BLW and BCS significantly during the metabolic trial, but Control group did not (Table 2). There were no significant differences in BLW between groups during the corpus luteum assessment (*p* > 0.05). The diameter of the preovulatory follicle at the time of GnRH treatment was similar between groups: 8.4 ± 0.7 and 10 ± 0.8 mm for Restricted and Control groups, respectively (*p* = 0.9). Similarly, the proportion of ovulated animals (7/7 and 7/7, *p* = 0.9) was not different between Restricted and Control groups, respectively. There was an effect of treatment (*p* = 0.01) and day (*p* = 0.001) but not interaction (*p* = 0.5) on CL diameter between groups. Diameter of CL was greater in the Control than that of the Restricted group at Days 5 and 7 after GnRH treatment (Figure 2A). Maximum CL diameter was lower (*p* < 0.04) in the Restricted (10.2 ± 0.6) than that of the Control group (12.1 ± 0.6 mm). Maximum CL vascularization was not different between Restricted and Control groups (0.37 ± 0.26 vs. 0.42 ± 0.25 cm^2^, respectively; *p* = 0.3, Figure 2B). However, there was an effect of time on CL vascularization (*p* < 0.001). Plasma progesterone and leptin concentrations were not affected by the treatment (*p* = 0.7 and *p* = 0.7, respectively, Figures 2C,D). Daily mean values of plasma leptin concentration during the luteal phase ranged from 6.9 ± 1.7 to 8.0 ± 1.6 ng/ml in both groups. There was no correlation between plasma leptin concentration on CL diameter, vascularization area, and plasma progesterone concentration (*p* > 0.05). The diameter of CL was positively correlated with vascularization area (*r* = 0.6; *p* < 0.001) and plasma progesterone concentration (*r* = 0.3; *p* < 0.01).

**Table 2:** Values (Mean ± SEM) of body live weight (BLW) and body condition score (BCS) of alpacas fed 40% of maintenance energy requirements (restricted group; *n* = 7) or fed *ad libitum* (control group; *n* = 7) before and after the nutrition management for 21 days.

**Variable**	**Control group**	**Restricted group**	***P*-value**
**Before**			
BLW (kg)	50.5 ± 2.6	52.6 ± 1.8^a^	0.3
BCS (1-5)	3.1 ± 0.2	3.1 ± 0.2^c^	0.9
**After**			
BLW (kg)	51.1 ± 2.5	49.3 ± 1.8^b^	0.2
BCS (1-5)	3.1 ± 0.2^x^	2.6 ± 0.2^dy^	0.002

^a, b^ different superscripts for BLW mean values differed within the group (P < 0.01).

^c, d^ different superscripts for BCS mean values differed within the group (P < 0.001).

*^x, y^ different superscripts for BCS mean values differed between groups (P < 0.01)*.

## Discussion

Based on the results of the present study, low energy restriction at 70% MER in llamas did not affect BLW, BCS, plasma leptin concentration, and metabolic parameters that may indicate a negative energy balance (Experiment 1). Moderate energy restriction at 40% MER in alpacas only reduced BCS and CL diameter, but no changes were detected in CL vascularization and plasma progesterone and leptin concentrations (Experiment 2).

Several studies have shown that energy status affects fertility in ruminant herds (27, 28). It has been described that nutritional restriction caused the absence or dysfunction of GnRH and gonadotrophic hormones secretion in sheep and cows (29–31). Hormonal dysfunction can result in the formation of a dominant follicle of smaller size and lower secretion of estradiol affecting estrus behaviors and the ovulatory process in cows (32, 33). Moreover, nutritional restriction reduced the diameter of the corpus luteum resulting in a lower production of progesterone which is associated with a higher rate of embryonic mortality and implantation /conception failures (32, 34). Negative energy balance has been associated to a dysfunction of various hormones and neuropeptides such as leptin, IGF-1, insulin, and neuropeptide Y that act at hypothalamic-pituitary-ovarian axis modulating GnRH secretion (11, 35).

In the present study, nutritional restriction at 70% MER for 28 days failed to induce negative energy balance in llamas (Experiment 1). However, females from the Restricted group lost 6% of their BLW; this loss can be partially attributed to a reduced gastrointestinal content that represents up to 11% of the BLW in llamas (36). Similar to our results, Bengoumi et al. (37) did not find any differences on body mass, hump volume, and lipid content after 2 months of restricted feeding at 68% MER in *Camelus dromedarius*. It has been described that the dromedary camel has the capability to reduce their energy expenditure up to 21% under fasting conditions (38). Llamas from the Restricted group maintained the homeostasis of proteins, lipids, carbohydrates, and energy; the values of metabolic markers were according to the referential values reported for camelids (39, 40). Negative energy balance (NEB) increased plasma concentrations of NEFA, BHB, or urea in previous studies conducted in llamas, alpacas, and vicuñas (17, 23, 41, 42). Starvation or dietary insufficiency results in mobilization of adipose stores, characterized by an increase in blood NEFA (over 0.6 mmol/L), and a mild increase in blood BHB (over 0.1 to 0.19 mmol/L) in camelids (43, 44). NEB induces amino acid breakdown to be used as energy substrate and possible gluconeogenesis that increases circulating urea (1, 17). The NEFA, BHB, and urea values reported in the study were lower than those cited before.

Sheep under energy diet restriction of 82% MER suffered a negative energy balance at Day 30 (45); apparently there must be a specific species response to the dietary restriction that also could be associated to the physiological and productive status of the animal.

In Experiment 2, although, alpacas fed 40% MER reduced their BLW up to 8%, it did not differ from the Control group. Nonetheless, BCS in alpacas from the Restricted group was lower than that observed in the Control Group indicating that negative energy balance caused lipolysis of fat reserves. Chagas et al. (35) suggested that measurement of BCS, unlike BLW, minimizes the influence of body size and gastrointestinal content. Leptin regulates energy balance, and its plasma concentration level correlates with fat reserves in several species (46) including BCS in llamas [*r* = 0.8, (23)]. Leptin exerts its effects through the leptin receptor (OB-R) which is highly expressed in the hypothalamus, where it is primarily responsible for suppression of food intake and stimulation of energy expenditure (47). Nonetheless, the restricted period of 21 days was not enough to induce some changes in leptin concentration as mean values observed by Day 1 (Day 0 = GnRH treatment) were similar between groups (7.6 ± 1.5 vs. 8.0 ± 1.5 ng/ml for Restricted and Control groups, respectively, Figure 2D). Leptin values observed in the present study were lower than that previously reported (~12 ng/ml) during the luteal phase in alpacas submitted to a fasting period during the pre-ovulatory stage (20).

The diameter of the pre-ovulatory follicle was similar between groups, and their sizes are similar to the mean values reported for non-pregnant, non-lactating alpacas (48). CL diameter in the Control group was significantly greater than that of the Restricted group. Indeed, CL diameter in the Restricted group was smaller than mean values (12–14 mm) reported in another study conducted in camelids (49). Corpus luteum formation involves the luteinization of granulosa and theca cells, increment of the ovarian blood flow, and the stimulation of a proliferation and differentiation process of endothelial and steroidogenic cells (50, 51). Luteinizing (LH) and growth hormones (GH) are the main luteotrophic hormones in bovines and along with insulin-like growth factor I (IGF-I) promote angiogenesis, mitosis, and progesterone production (52). Taking into account that 85% of the cells that proliferate in a developing CL are endothelial cells, it could be speculated that the greater size of CL observed in the Control group could be attributed to an increase of CL vascularization area (52), but although CL vascularization area was numerically greater in the Control than that of the Restricted group, these were not significantly different and so failed to explain CL size differences. The vascularization values observed in the present study were similar to those reported in other studies in alpacas and llamas (20, 25, 26).

It is well-known that negative energy balance (NEB) reduces systemic LH secretion in ruminants influencing CL development and progesterone production (32, 53). NEB also negatively affects the plasma concentration of IGF-I, a hormone that stimulates LH secretion at the hypothalamic and pituitary levels (11, 54). In addition, Insulin-like growth factor receptor has been detected in most of the luteal cells in alpacas suggesting that IGF-I may be involved in the CL steroid synthesis in camelids (16). It has been described that LH and IGF-I hormones also act synergistically stimulating the proliferation and differentiation of granulosa cells, as well as promoting steroidogenesis, vascularization, and oocyte health (16, 55). Nevertheless, the diameter of the pre-ovulatory follicles as well as the ovulatory rate of the Restricted alpacas were not affected by NEB, which rules out a dysfunction of these hormones.

Despite the differences in CL diameter between groups, progesterone concentration was similar in both Restricted and Control groups; these values were similar to those reported in other studies in alpacas (56, 57). A positive correlation between CL vascularization and plasma progesterone concentration has been reported in camelid studies (20, 25). Then, the stability of the leptin plasma concentration of the Restricted group could stimulate an adequate vascularization of the CL to provide the precursors needed for progesterone production (19, 20).

The CL diameter and plasma progesterone concentration were positively correlated in the present study. Probably, a higher energy challenge could affect both parameters simultaneously, similarly as described in restricted llamas that lost near to 20% of BLW (17). Similarly, in ruminants, a decline in CL diameter and progesterone plasma concentration was observed in the two estrous cycles preceding anovulation, in heifers that lost 22% of BLW (53).

In ruminants, diets 40% MER for 14 days reduced leptinemia in sheep (58), and 60% MER for 21 days did it in a group of cows when compared to 130% MER (59). In addition, dietary restriction of 40% MER during 15 or 21 days induced anestrus in 60% of heifers, with reduced pre-ovulatory follicles or CL (29, 34). In this sense, the ability for camelids to maintain the energy balance and leptinemia may have comparative advantages of these species with respect to ruminants in terms of reproductive performances in the High Andes. The differential response between ruminants and camelids under nutritional restriction could be based on the following: (i) camelids are more efficient in reducing their basal metabolic rate during fasting (38), (ii) apparently, camelids use, to a greater extent, proteins as substrate for energy production and gluconeogenesis, being more efficient with the urea recycling (1, 7, 43).

We conclude that low energy restriction at 70% MER during 28 days in llamas did not affect BLW, BCS, plasma leptin concentration, and metabolic parameters that may indicate a negative energy balance (Experiment 1). The moderate energy restriction at 40% MER during 21 days in alpacas reduced BCS, BLW, and the CL diameter, but no changes were detected in CL vascularization, plasma progesterone, and leptin concentrations (Experiment 2). The results of the present study suggest that these species must be submitted to longer periods of nutritional restriction to induce a significant effect on systemic leptin concentration in order to impair follicular growth, CL vascularization, and progesterone production.

## Data Availability Statement

The raw data supporting the conclusions of this article will be made available by the authors, without undue reservation.

## Ethics Statement

The animal study was reviewed and approved by Bioethic Committee from Universidad Catolica de Temuco and Universidad Austral de Chile.

## Author Contributions

CN design the study and execute all the experiments, participate in data collection, data interpretation and writing the manuscript. FH, JA,GS, and ST collaboration in saplles collection, ultrasonography, and animal management. MR experimental design and writing of the manuscript, and direction of the study. All authors contributed to the article and approved the submitted version.

## Conflict of Interest

The authors declare that the research was conducted in the absence of any commercial or financial relationships that could be construed as a potential conflict of interest.
